# ‘Just knowing it’s there gives me comfort’: Exploring the benefits and challenges of autism alert cards

**DOI:** 10.1177/13623613241286025

**Published:** 2024-10-18

**Authors:** Chris Edwards, Abigail MA Love, Rebecca L Flower, Ru Ying Cai, Vicki Gibbs

**Affiliations:** 1 Autism Spectrum Australia, Australia; 2 Griffith University, Australia; 3 La Trobe University, Australia; 4 The University of Sydney, Australia

**Keywords:** autism, autism alert card, autism ID card, Autistic identity, communication tool, disclosure

## Abstract

This study explores the experiences and outcomes associated with the use of an autism alert card, developed to assist with the complexities of autism disclosure. A mixed-methods approach was employed, surveying 272 Australian participants, including 136 Autistic adults, 128 parents or caregivers of Autistic children and eight Autistic children. Our research aimed to understand who is using the alert card, in what contexts and the differences in perceptions of the card between Autistic individuals and their parents. Findings indicate that about half of the participants used the card, primarily in public settings such as transport (17%), healthcare (16%) and retail (16%). The card was seen as a valuable tool for facilitating communication, reducing the need for verbal explanations and providing a sense of security. However, challenges were reported, including societal disbelief, patronising attitudes and internalised stigma, highlighting the need for greater public education on autism. Despite these challenges, majority of participants (76.2%) would recommend the card. This research underscores the dual nature of autism alert cards as both beneficial and limited by societal attitudes, emphasising the need for comprehensive training and awareness programmes to maximise their effectiveness.

**Lay abstract:**

This study looks at how people use and feel about autism alert cards, which help Autistic people communicate their diagnosis/identity. We surveyed 272 Australian people, including 136 Autistic adults, 128 parents of Autistic children and eight Autistic children. About half of the participants used the card after ordering it, mostly in public places such as public transport, healthcare settings and in retail settings. People found the card helpful because it made it easier to explain their needs without having to speak and provided them a sense of security. However, some people treated Autistic people poorly after being shown the card due to a lack of understanding about autism. Many participants felt that more education about autism is needed to improve how people react to the alert card. Despite these challenges, most participants (76.2%) would recommend the alert card to others. This research shows that while autism alert cards can be very helpful, their effectiveness depends on how well other people understand and accept autism. To make these cards work better, we need more training and awareness programmes for the general public and professionals such as doctors or police officers who may interact with Autistic people.

Disclosing, or sharing one’s Autistic identity,^1^ can be fraught with complexity, and can affect the lives of Autistic individuals across numerous social and professional contexts (Edwards et al., 2023; Romualdez et al., 2021). While disclosure can lead to appropriate support and increased understanding, it also carries the risk of negative outcomes, such as exposure to stigma and discrimination (Botha et al., 2022; Farsinejad et al., 2022; Huang et al., 2022; Lindsay et al., 2021; Thompson-Hodgetts et al., 2020). These adverse experiences can include infantilisation, loss of relationships, loss of jobs and living in fear regarding disclosure (Edwards et al., 2023). In addition, negative experiences of disclosure can also lead to internalised stigma for Autistic people, harming their self-worth and well-being (Han et al., 2021). Given these challenges, there is a clear and pressing need to support Autistic people in navigating the intricacies of autism disclosure, preparing them for the disclosure itself but also for the reactions of others (Han et al., 2024; Love, Edwards, et al., 2023; Thompson-Hodgetts et al., 2020).

Methods of disclosure can be verbal (e.g. conversation), non-verbal (e.g. email), or a combination of both. Numerous disability and advocacy organisations worldwide have developed and promoted a range of tools to facilitate disclosure of autism, such as identification cards, bracelets, health passports, and autism alert cards (sometimes used interchangeably with identification cards). These tools are designed to communicate that the person is Autistic, and may also include varying levels of information about the individual, about autism in general, or some suggested accommodations and supports. Despite their widespread promotion and availability, emerging research indicates a preference for verbal communication to disclose rather than the use of tools such as identification cards or jewellery (Love, Cai, et al., 2023; Love, Edwards, et al., 2023). However, the preference for verbal over non-verbal communication is not universal and can vary depending on the specific context and whether the individuals are known or unknown to the Autistic person (Howard & Sedgewick, 2021).

Autism alert cards are among the most accessible disclosure tools and are extensively available worldwide to assist Autistic individuals or parents and caregivers during a disclosure experience. For instance, in the United Kingdom, the National Autistic Society (n.d.) provides cards that offer educational and personal information in formats accessible to Autistic persons, their parents and supporters. Similarly, in Australia, the Autism Association of Western Australia (n.d.) distributes cards to assist Autistic individuals during emergencies, particularly in interactions with police services where it has been suggested that disclosure may help facilitate better communication and understanding (Allely & Murphy, 2023; Maras & Bowler, 2014; Richardson et al., 2016). In addition, as highlighted by MacLennan et al. (2023), sensory experiences in public spaces such as transport, retail and healthcare can be disabling for Autistic individuals, suggesting that autism alert cards could also be useful in these contexts where verbal communication may be difficult. However, research on their use in these critical and everyday interactions is limited (Logos et al., 2021), and little is known about the perspectives of Autistic individuals regarding these tools.

Three studies have specifically evaluated the usefulness of autism alert cards. In the first study, Austin et al. (2016) asked parents to complete a survey that included a vignette depicting a parent and child during a crisis (e.g. meltdown), where participants either received disclosure via an alert card or no disclosure at all. Participants in the non-disclosure condition rated the parent more negatively and experienced more negative reactions compared to those who received an autism alert card. In a follow-up study, Austin et al. (2018) expanded on this research by comparing the effectiveness of two different disclosure methods: an autism alert card and a disclosure bracelet. Both disclosure methods significantly reduced negative perceptions compared to the non-disclosure condition, with the alert card being slightly more effective. In addition, Logos et al. (2021) found that providing an autism information card during police–suspect interrogations significantly attenuated biased evaluations and judgements of guilt. These findings suggest that autism alert cards can mitigate negative perceptions and unfair judgements, although their effectiveness in real-world settings and various contexts needs further exploration.

Notwithstanding the lack of scientific exploration of the effectiveness of autism alert cards, public perception drawn from social media content is generally positive (Davis, 2021). Community members believe that these cards can help bridge the communication gap between Autistic and non-Autistic people. However, there are also concerns that they could also lead to further discrimination (Davis, 2021). These concerns are warranted, as studies show that a well-informed understanding of autism on the part of the person being disclosed to can positively impact the disclosure experience, while a stigmatised view may exacerbate challenges (Morrison et al., 2019; Romualdez et al., 2021; Sasson & Morrison, 2019). In addition, there is a fear that in high-pressure situations, such as during interactions with law enforcement, reaching for an identification card could be misinterpreted as a threatening gesture, potentially leading to dangerous outcomes, including the possibility of being shot (Davis, 2021). Furthermore, in emergency services contexts, the practicality of such engagement is often compromised by time constraints, which may not always allow for thorough understanding (Logos et al., 2021). This is problematic given the recognised need for autism-related education and training for professionals in such contexts (e.g. Corden et al., 2021; Sreckovic et al., 2023). Therefore, while the educational content on an alert card is inherently valuable, its effectiveness may largely depend on the recipient’s readiness and ability to engage meaningfully with the provided information. This readiness is deeply reliant on society and the cultural context surrounding autism acceptance, as the card’s utility hinges on the recipient’s basic understanding of autism and their willingness to respond appropriately. Small but significant changes in the way we talk about autism, such as adopting neuro-affirming language (Bottini et al., 2024), may help increase acceptance and understanding, thereby enhancing the likelihood that individuals will respond appropriately to gestures of disclosure.

## This study

Despite their widespread use and perceived value, there is a notable gap in the literature concerning personal experiences with autism alert cards and perspectives regarding their effectiveness. This oversight presents a critical area for research, requiring the inclusion of both Autistic and non-Autistic people (e.g. parents of Autistic children) given their differing disclosure experiences (see Thompson-Hodgetts et al., 2020 for review). Our study targeted participants who requested an alert card from an Australian national autism-specific service provider. The creation of the card was led by Autistic individuals. The card featured a tri-fold design. On the front, the card prominently displayed the notice ‘ATTENTION I am Autistic’. Upon opening, the card outlined information about autism and provided guidance on how to support an Autistic person, such as during meltdowns. In addition, the card included designated spaces for the individual to write their name and emergency contact details (see Supplementary materials for images of the card). Given the identified gaps, this research aims to explore the experiences and outcomes associated with this specific autism alert card by addressing the following questions:

1. Who is using the autism alert card and in what contexts?
2. Are there any differences in the frequency of usage, reactions and endorsement of the card between Autistic people and parents/carers?
3. What are the experiences and impacts of having and using the alert card from the perspectives of Autistic individuals and parents/carers?

## Method

### Participants

The sample comprised 272 participants, including 136 Autistic adults and 128 parents or caregivers, along with eight Autistic children (see Table 1). Due to the limited number of responses directly from Autistic children and as our second research question requires a comparison of responses from Autistic people to parents/carers, their data were combined with Autistic adults to form an ‘Autistic individuals’ group. Respondents were drawn from various locations across Australia, with more than half indicating residency in a capital city (57.0%; *n* = 155), followed by 21.0% in a regional city (*n* = 56), 13.5% in a country town (*n* = 36) and minimal participants selected ‘other’ (*n* = 16) or a regional property (*n* = 4). The majority of Autistic participants (93%) reported a formal autism diagnosis, with the remainder (*n* = 10) self-identifying as Autistic. Autistic individuals, based on self-reports and reports from parents, experienced a range of co-occurring conditions including anxiety disorder (57.4%, *n* = 156), attention-deficit/hyperactivity disorder (ADHD; 50.4%, *n* = 137), mood disorder (18.4%, *n* = 50), sleep disorder (16.5%, *n* = 45), intellectual disability (15.4%, *n* = 42), eating disorder (13.2%, *n* = 36), personality disorder (5.1%, *n* = 14) and other co-occurring neurological and physical conditions (e.g. epilepsy, Tourette syndrome; 29.0%, *n* = 79).

**Table 1.** Participant demographic information (*N* = 272).

	Autistic individuals *n* = 144	Parent/carers *n* = 128
Mean age (years)	37.4 (*SD* = 15.4)	45.5 (*SD* = 10.7)
Gender		
Male	39 (27.1%)	13 (10.2%)
Female	76 (52.8%)	114 (89.1%)
Non-binary/Transgender/Other	27 (18.8%)	0 (0.0%)
Rather not say	2 (1.4%)	1 (0.8%)
Ethnicity^a^		
Aboriginal Australian	6 (3.8%)	12 (9.0%)
Torres Strait Islander	0 (0.0%)	1 (0.7%)
Pacific Islander	2 (1.3%)	1 (0.7%)
Asian (South Asian, South East Asian, Chinese)	5 (3.2%)	10 (7.5%)
Middle Eastern	0 (0.0%)	2 (1.5%)
African	1 (0.6%)	0 (0.0%)
Hispanic	2 (1.3%)	3 (2.2%)
White	117 (74.5%)	89 (66.4%)
Other	17 (10.8%)	12 (9.0%)
Rather not say	7 (4.5%)	4 (3.0%)
Education		
Primary school	4 (2.8%)	1 (0.8%)
High school	25 (17.6%)	18 (14.4%)
Vocational education and training	46 (32.4%)	47 (37.6%)
Higher education	52 (36.6%)	52 (41.6%)
Other	14 (9.9%)	3 (2.4%)
Rather not say	1 (0.7%)	4 (3.2%)

a Participant could select multiple responses.

In addition to participant demographics, background information was also collected from the parents/caregivers’ group about their Autistic children. The average age of their children was 14.4 years (*SD* = 6.9 years, range 2–33 years). Their gender distribution included 80 males (65.6%), 38 females (31.1%) and four non-binary/transgender/other (3.3%). The majority (77.9%, *n* = 95) were attending early childhood or school settings.

### Measure

We designed a survey that started by offering participants the choice between an Easy Read or standard Participant Information and Consent Form, to increase the accessibility of our questionnaire. Upon consenting, participants answered background questions about themselves, and parents or caregivers also provided information about their Autistic child. To further support accessibility, the online survey incorporated basic visual supports such as emoticons. The survey included specific questions about the use of the autism alert card, such as where it is kept, whether it has been used, who primarily uses it, how often it is used and in what types of situations or settings it has been used (see Supplementary materials for survey questions). We also asked about the types of reactions encountered and the helpfulness of the card. Participants had multiple opportunities to provide open-text responses and suggest changes to make the autism alert card more applicable or helpful. A significant portion of participants (*n* = 246) provided at least one open-text response, with the total word count per participant ranging from 23 to 968 words, and an average of 171 words per participant. The average duration of completing the survey was 21 min.

### Procedure

This study received ethics approval from Griffith University Human Research Ethics Committee (2024/059). During the period between September 2020 and February 2024, a total of 6361 alert cards were distributed upon individual request at no cost to Autistic adults (*n* = 1957, 30.8%), parents or caregivers of Autistic individuals (*n* = 4039, 63.5%) and support workers or organisations (*n* = 365, 5.7%). As part of the distribution process, recipients were asked whether they would be willing to participate in a feedback study about their experiences with the alert card and 4682 indicated consent. Participants were contacted by researchers via email on three occasions in February and March 2024 and invited to participate in the online study.

**Figure 1.**
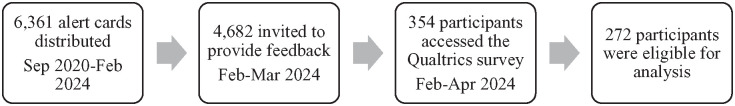
Participant flow diagram.

The following figure (see Figure 1) details our recruitment and data-cleaning procedure for this study.

Exclusion reasons included participants who did not complete the survey beyond providing their consent (*n* = 63), participants who did not complete consent (*n* = 12), three participants who provided misleading responses (e.g. repeatedly discussing irrelevant topics such as US presidents), two participants who were not a parent or Autistic person and two participants who did not reside in Australia. Due to the voluntary nature of data collection, some questions in the dataset were not answered by all participants.

### Data analysis

We used descriptive statistics to address the first research question concerning the usage and context of alert cards. For the second research question about differences in perception between Autistic people and parents, we conducted three chi-square tests for association to investigate the relationship between participant type (e.g. Autistic individual and parent/carer) and perceptions of the alert card (as measured by frequency, reactions and endorsement) because the data were categorical and did not meet the assumptions required for parametric tests.

For our third research question concerning the experiences of individuals who possessed and/or used the alert card, we applied the reflexive thematic analysis framework of Braun and Clarke (2006, 2013, 2019). We chose this method for its flexibility and depth, which permitted both semantic and latent coding. Semantic coding allowed us to interpret the explicit content of the responses, clarifying straightforward experiences with the alert card. Latent coding explored the subtler impacts of the alert card on social interactions and individuals’ sense of safety and autonomy. An Autistic researcher (C.E.), who was a late-diagnosed adult familiar with the complexities of autism disclosure and did not use an alert card, led and enriched this part of the analysis. Our team engaged in regular discussions to refine themes and ensure they authentically represented the participants’ experiences, supported by NVivo 14.

### Community involvement statement

The idea for the study was proposed by the senior author (V.G.) who is a non-Autistic clinical psychologist, autism researcher and parent of an Autistic young person. The study was led by an Autistic early career researcher (C.E.) who was formally identified as Autistic as an adult and does not have an autism alert card. They received close support from a non-Autistic early career autism researcher with a sibling who has an alert card (A.M.A.L.). The research team also included an ADHDer (R.F.) and a non-Autistic autism researcher (R.Y.C.). The composition of the team was communicated at the outset in the participant information sheet to ensure transparency. All team members have professional qualifications in education and/or psychology.

## Results

### Autism alert card usage

**Figure 2.**
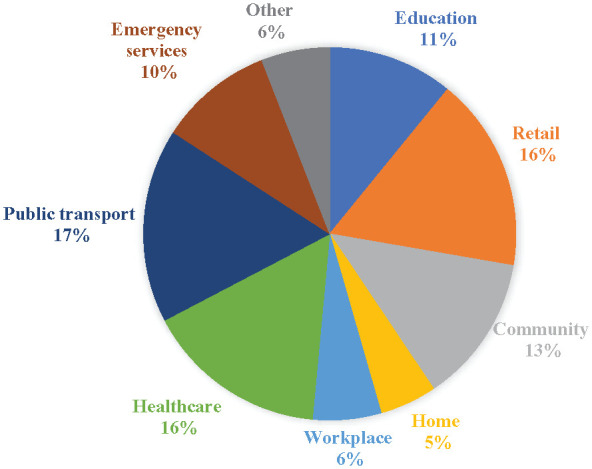
Percentage of autism alert card usage by context.

About half of the participants (Autistic, *n* = 78, 55.7% and parent/carer, *n* = 53, 44.9%) reported that they had used the card at least once since receiving it. For Autistic participants who requested the card (*n* = 78), the majority (*n* = 77; 98.7%) reported being the primary user of the card, with only one person indicating their parent/carer or support person was the primary user. For parent participants who requested the card (*n* = 53), just under half (*n* = 25; 47.2%) reported that they were the primary user and just over half (*n* = 28, 52.8%) indicated that their Autistic child was the primary user. Participants reported that the cards were typically being carried in a personal bag or wallet (*n* = 205, 71.9%), while participants less frequently reported that it was kept at home (*n* = 32, 11.2%), in a vehicle (*n* = 14, 4.9%) or in another location (*n* = 34, 11.9%). Finally, Figure 2 shows the contexts where the cards were used, with the most frequent usage being public transport, healthcare and retail locations.

### Autism alert card perceptions

We gathered further information from participants who reported that the alert card had been used (*n* = 131) to understand more about the frequency of usage and the reactions they received. The majority of participants (*n* = 75, 57.3%) indicated that the card had been used ‘rarely’ while 32.1% (*n* = 42) stated that it had been used monthly or weekly. Approximately 10.7% (*n* = 14) stated it was used daily. With regard to reactions received, 70.5% (*n* = 86) reported a majority of positive reactions and 9.0% (*n* = 11) reported mostly negative reactions. About one in five (20.5%, *n* = 25) reported mixed reactions.

Endorsement of the autism alert card was measured across the majority of the sample (*n* = 235) and indicated that just over three quarters (76.2%, *n* = 179) would recommend the card to Autistic individuals and/or their family members, 18.7% (*n* = 44) stated they might recommend it and only 12 participants (5.1%) indicated they would not recommend the card. There were no significant associations between participant type (Autistic individual and parent/carer) and perceptions of the alert card for all three variables: frequency, χ^2^ (3, *n* = 131) = .66, *p* = .882; reactions, χ^2^ (2, *n* = 122) = 1.23, *p* = .540; and endorsement, χ^2^ (2, *n* = 235) = 2.31, *p* = .315.

### Autism alert card experiences

**Figure 3.**
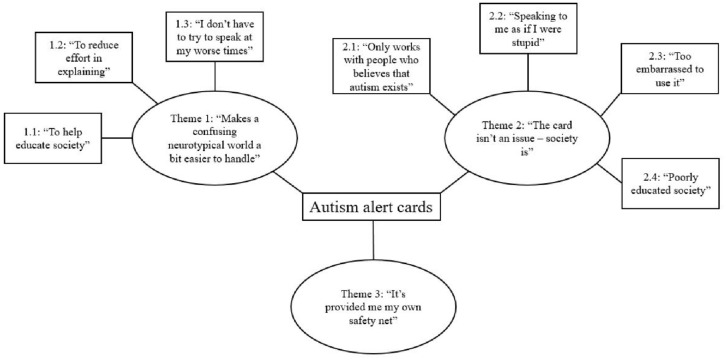
Thematic map of experiences with an autism alert card.

We further explored the experiences of participants with the alert card to better understand how the card may impact the lives of Autistic individuals and those around them (research question three). We generated themes that illustrated both positive and negative experiences associated with use of the alert card, including the broader societal aspects that impact how these encounters play out. Below, we present a thematic map (see Figure 3) that outlines these themes, followed by detailed discussions of each theme with quotes including the participant type and ID number (e.g. Autistic adult_18).

### Theme 1: ‘Makes a confusing neurotypical world a bit easier to handle’

Theme 1 encapsulates how the alert card served as a tool for bridging understanding between Autistic people and broader society. This theme is split into three subthemes, each describing different ways in which participants detailed how the card provided education, reduced the need for verbal explanation and helped them feel supported during challenging interactions.

#### Subtheme 1.1: ‘To help educate society’

Participants reported that using the alert card helped improve understanding and facilitate interactions between Autistic people and wider society. Particularly, it served as an effective tool for educating others about autism, communicating needs clearly and fostering greater awareness. For example, one parent highlighted the educational impact of the card: ‘people were often surprised about what autism is exactly and commented how good the card is in educating people about Autistic behaviours’ (parent/caregiver_240). This sentiment was echoed by Autistic participants, including an adult who found that the card prevented potential misunderstandings and enhanced their interaction with healthcare providers: ‘I have a fear of dentist offices and procedures. First time I went to a new dentist, I couldn’t talk, but showing the card allowed the receptionist to ask the needed questions differently to get me checked in’ (Autistic adult_18).

Some participants felt that the card served as a straightforward tool for explaining autism and led to practical adjustments in the user’s favour. For example, Autistic adults appreciated how the card helped overcome challenging situations such as engaging with a Protective Service Officer who misunderstood sensory behaviours on public transport, ‘but changed their behaviour to a more supportive one once the card was shown’ (Autistic adult_228). From a parental perspective, the card proved invaluable in educating others and gaining empathy for their children, as one parent noted it ‘assisted others to become more comfortable and have a greater understanding of Billy [pseudonym] and his needs and behaviours’ (parent/caregiver_84).

#### Subtheme 1.2: ‘To reduce effort in explaining’

Many participants found their alert card helped reduce the effort needed to explain autism-related needs, particularly in scenarios where verbal communication was challenging or impractical. These cards enabled a swift, non-verbal disclosure in both everyday interactions and more stressful environments. As one Autistic adult explained, ‘there are occasions when time is short & verbal explanations [are] not useful’ (Autistic adult_15). The simplicity and immediacy of the card were further exemplified by its use in social settings: ‘it is a quick, non-verbal explanation on why she can’t speak to strangers, why she is a little different from others’ (parent/caregiver_215). This allowed participants to maintain their privacy and dignity in public spaces, mitigating potentially uncomfortable situations quickly and discreetly. In addition, the card served a similar purpose in broader social gatherings, as ‘it is an easy non-verbal way to explain why we need extra support or compassion when shopping or attending events’ (parent/caregiver_97).

#### Subtheme 1.3: ‘I don’t have to try to speak at my worse times’

Several Autistic adults acknowledged the value of having an alert card in moments of overwhelm such as sensory overload or engaging with emergency services. These cards help take ‘the pressure off trying to explain your situation when you can’t even speak’ (Autistic adult_156). For example, in response to a drug test at a music festival, ‘it helped make the police less assertive and more relaxed towards me’ (Autistic adult_199). The card also facilitated smoother interactions in other stressful environments as one participant said, the card ‘helps me to get through security in airports, and alert police that I may be nonverbal and physically twitchy when stressed’ (Autistic adult_39). Moreover, participants consistently shared that the card supported them in daily situations that could provoke anxiety, such as ‘in situations when I’m out in public and get overwhelmed and become situationally mute. This happens in shops, airports, healthcare settings, etc’ (Autistic adult_102).

### Theme 2: ‘The card isn’t an issue – society is’

While Theme 1 highlighted participants’ positive perceptions of the card’s use in everyday and critical situations, many participants also shared negative experiences with the alert card that they attributed to broader society rather than the design of the card (e.g. ‘I don’t think it’s the “card” itself that is unhelpful, but the people viewing the card’; Autistic adult_33). Theme 2 is broken down into four subthemes that relate to these external factors such as societal disbelief, patronising attitudes, and stigma that affect the card’s utility and the autonomy of Autistic individuals.

#### Subtheme 2.1: ‘Only works with people who believe that autism exists’

This subtheme describes how the effectiveness of the alert card was frequently constrained by societal disbelief or ignorance about autism. Users often faced scepticism that they felt significantly undermined the card’s utility. For instance, one participant highlighted experiences where ‘people have called it a fake card or [suggested] I’m faking autism’ (Autistic adult_16). Participants reported that the utility of the alert card was further compromised by the lack of awareness and respect from authoritative figures, particularly in interactions with law enforcement:

I informed officers several times I had autism and presented the autism alert card. Officers had a quick ‘glance’ at the card, but did not read it and gave it back. I was told ‘we will deal with that later’. (Autistic adult_33)

Such disregard in one context prompted further scepticism from participants about the general effectiveness of the card, as one Autistic adult questioned, ‘police officers have no idea or care what it [card] meant so why would anybody else take notice?’ (Autistic adult_193).

#### Subtheme 2.2: ‘Speaking to me as if I were stupid’

This subtheme describes how participants felt that alert cards led to infantilisation in some instances, where people who were shown the card simplified their communication excessively or misjudged the Autistic person’s intellectual abilities. For example, one participant recounted experiences in medical settings:

Some people don’t even take the time to read it or blatantly just fob it off after reading it and come to the conclusion that I am intellectually disabled, not high functioning, or that I am mentally ill. This unfortunately happens most in hospitals. (Autistic adult_181)

These experiences were common in interactions with the healthcare and justice systems:

I was extremely anxious about this [test] and had trouble communicating so I handed the card to the male [practitioner]. He responded by raising his voice and speaking at me as if I were stupid. He also told me that I couldn’t expect everyone to meet my needs. I fled. (Autistic adult_247)

During encounters with law enforcement, the misunderstandings can be particularly pronounced, as another cardholder experienced:

During COVID restrictions I couldn’t wear my mask as I feel like I was suffocating . . . I was in a service station and got stopped by a police officer. I showed my card and he belittled me, I burst into tears. (Autistic adult_193)

#### Subtheme 2.3: ‘Too embarrassed to use it’

Some participants were reluctant to use the alert card due to embarrassment and the stigma associated with disclosing one’s Autistic identity. The feelings of shame and the societal pressures that accompany them often deterred individuals from using the card, even when it could aid their interaction with others. For instance, one individual reflected on their internal conflict:

I think I haven’t used it with others probably because I don’t think of it at the time due to internalised ableism and shame about being Autistic, which I haven’t quite accepted yet. Masking is so entrenched that I don’t know if I could ever show a card that has ‘I’m Autistic’ in giant letters. (Autistic adult_263)

Another person stated that they have not used the alert card because of ‘embarrassment and shame’ (Autistic adult_95). Parents also reported similar observations about their children, noting their ‘son feels too embarrassed / stigmatised to produce it’ (parent/caregiver_26). The challenge extended to public situations where the potential for misunderstanding was considered high such as when travelling alone on planes:

I did not use the card to explain why I was crying, very restless and walking up and down the aisle a few times because I didn’t have any confidence that they would understand and trust it at face value. . . So, when I’m at my most vulnerable I cannot afford a response based in stigma. (Autistic adult_131)

#### Subtheme 2.4: ‘Poorly educated society’

Many participants highlighted the limited impact of the alert card due to a general lack of understanding about autism within the community. A common frustration shared by participants was that, ‘this card does nothing–the majority of people have no clue about ASD or the massive range of behaviours’ (parent/caregiver_271). The disparity in public reactions to the card led to some participants calling for increased awareness: ‘once people see or notice the card on my child or question the way in which they act, it’s quite a different response to how they see them, we do not have enough education in the community sadly’ (parent/caregiver_43). Moreover, there was a call for more proactive information dissemination that does not solely rely on Autistic people: ‘it [card] has helpful information but I think people just need to be more informed without it requiring Autistic people to do so’ (Autistic child_146). An Autistic adult summed up the situation by emphasising the lack of ‘visibility & awareness in a poorly educated society that pretends that we are not present & do not exist in the society’ (Autistic adult_276).

### Theme 3: ‘It’s provided me my own safety net’

While many participants expressed challenges with societal responses and the limitations of the alert card as discussed in Theme 2, Theme 3 highlighted the personal empowerment and security that the card provided for many participants, providing a vital safety net. The presence of the card alone offered reassurance, as one user expressed, ‘just knowing it’s there gives me comfort’ (Autistic adult_65). This sentiment was echoed by another who appreciated the ‘peace of mind it gives me knowing I can use it if need be’ (Autistic adult_156). These statements highlighted how even the mere possession of the card could alleviate anxiety, ensuring that individuals felt prepared and secure, ready to navigate social interactions more confidently.

Furthermore, parents described how the card acted as a silent advocate, valued in potentially overwhelming or in emergency situations. The card offered reassurance regarding their children’s safety and ability to communicate in public spaces such as one parent who noted, ‘it’s been a safety barrier for my son’ (parent/caregiver_7). Another parent shared the specific benefit for their son who had previously had a distressing encounter:

My son had a negative experience with public transport ticket inspectors and now with the card feels that he has a way to explain himself if he encounters a similar situation. He feels safer out in public on his own and I feel reassured that he has this card to communicate his needs if he requires. (parent/caregiver_27)

## Discussion

This study aimed to bridge a gap in the literature regarding the use and impact of autism alert cards, a widely available yet understudied tool for assisting with autism disclosure. Our findings revealed that approximately half of the participants who received a card used it and the majority of participants reported positive experiences when using the card. These predominantly positive experiences align with the public perception of the use of autism alert cards (Davis, 2021). Importantly, participants shared with us the intrinsic value of simply possessing the card, whether it was used or not. This supports the notion that autism alert cards can be valuable tools for facilitating disclosure, echoing the identified need for such tools in previous research (Han et al., 2024; Love, Edwards, et al., 2023; Thompson-Hodgetts et al., 2020).

Our study found that autism alert cards are most frequently used in public settings such as transport, healthcare and retail environments, as well as during interactions with emergency services. These contexts often present unique challenges for Autistic individuals, making the alert card a valuable tool for facilitating communication and understanding. Participants reported that the card helped bridge understanding between Autistic individuals and broader society, supporting smoother and more empathetic interactions. These findings provide initial evidence supporting claims regarding the potential helpfulness of autism alert cards (Allely & Murphy, 2023; Doherty et al., 2021; Maras & Bowler, 2014; Richardson et al., 2016). Furthermore, our findings support the observations of MacLennan et al. (2023), who noted that sensory experiences in public spaces can be particularly disabling for Autistic individuals. According to our participants, the use of autism alert cards in these contexts appears to help by facilitating quicker and more effective communication about the individual’s needs, thus potentially reducing the sensory overload and stress experienced in such environments or providing a means of communication when verbal communication is not an option due to overload.

However, consistent with previous disclosure research (Edwards et al., 2023; Love, Edwards, et al., 2023), many of the negative experiences reported by participants in this study were discussed in relation to society’s limited knowledge and understanding of autism. As a stigmatised view of autism can increase challenges following disclosure (Morrison et al., 2019; Romualdez et al., 2021; Sasson & Morrison, 2019), it is hoped that the informative material provided on an autism alert card mitigates some of this risk. However, participants in our sample consistently shared that people, often in authority roles (e.g. police and healthcare), ignored or dismissed the information contained in the card. While it is understandable that time constraints associated with these roles may limit their ability to read the information in full (Logos et al., 2021), it is disappointing that our participants were responded to with dismissive or infantilising behaviour on occasion, particularly at times when Autistic people may be at their most vulnerable and are offering a communication tool or gesture of understanding.

The challenges associated with autism alert cards are not unique and mirror those faced by other communication tools such as autism health passports (Ellis et al., 2023; Grant et al., 2024). Both tools are designed to improve interactions between Autistic individuals and professionals, yet their potential is often undermined by insufficient engagement and a lack of autism-specific training among professionals. Without the necessary understanding and training, these tools risk failing to provide the intended support, leading to reluctance among Autistic individuals to use them due to fears of stigma and dismissal (Grant et al., 2024). These findings highlight the urgent need for systemic change, including comprehensive training, greater awareness and the meaningful involvement of the Autistic community in the development and implementation of such tools.

### Practical implications

Given that the majority of participants experienced mostly positive reactions when using the autism alert card, found it helpful and would recommend it to others, the promotion of these cards should continue within the Australian community. The demonstrated benefits emphasise the card’s value as a tool for supporting Autistic individuals and their families. In addition, many participants reported that simply possessing the card provided a sense of safety and reassurance, even if it was never used. Therefore, ongoing efforts to distribute and educate the public about the use of autism alert cards are warranted to maximise their positive impact and facilitate smoother interactions in various public and professional settings.

Although the majority of autism alert card experiences were positive, the negative experiences continue to illustrate how little society understands about autism (per Edwards et al., 2023). The problematic interactions with police and healthcare professionals continue to highlight significant gaps in autism knowledge among these groups (Corden et al., 2021; Sreckovic et al., 2023). These findings underscore the urgent need for comprehensive, targeted training programmes that equip professionals with the skills to recognise autism, understand its diverse presentations, and respond appropriately to autism alert cards or other alternative forms of communication. Even without formal training, simply taking the time to read the card and follow its guidelines can significantly improve interactions with Autistic individuals.

### Limitations

While this study provides valuable insights, several limitations must be acknowledged. First, the study is based on data from Australian participants, focusing specifically on their experiences with using an autism alert card in Australia. As a result, these findings may not be generalisable to other contexts, where varying levels of autism acceptance or differing familiarity with the concept of an alert card could lead to different experiences. In addition, the study centres on one specific autism alert card within the Australian context, which may further restrict the generalisability of the results to other types of alert cards, even within Australia. Another limitation is the underrepresentation of Autistic children in our sample. Their experiences with autism disclosure are critically important yet often overlooked in research (Thompson-Hodgetts et al., 2020). Although we aimed to include their perspectives, the small number of participating children limited our ability to make comprehensive comparisons with the experiences of adults and parents/caregivers. Furthermore, our sample predominantly consists of White females, which may not fully capture the diverse experiences of the broader Autistic population. This demographic homogeneity limits the extent to which our findings can reflect the varied experiences of all Autistic individuals. Finally, a relatively small percentage of those who received the autism alert cards chose to complete the survey. While this response rate aligns with expectations for voluntary surveys, it may impact the generalisability of the results. The views and experiences captured in this study might not fully represent the broader population of autism alert card users, particularly those who opted not to participate.

## Conclusion

This study contributes to the understanding of autism alert cards as a valuable tool for facilitating communication and support for Autistic individuals. The findings accentuate the dual nature of autism alert cards: they can serve as powerful tools for facilitating disclosure and enhancing social interactions, yet their effectiveness is heavily contingent upon the knowledge and attitudes of those receiving the disclosure. Despite their potential, societal challenges and stigma remain significant barriers to the effectiveness of autism alert cards. By continuing to research and advocate for these tools, we can better support Autistic individuals in navigating disclosure and foster a more inclusive and understanding society. Through these efforts, we move closer to a world where Autistic people are not only accepted but embraced and understood in all aspects of life.

## Supplemental Material

sj-docx-1-aut-10.1177_13623613241286025 – Supplemental material for ‘Just knowing it’s there gives me comfort’: Exploring the benefits and challenges of autism alert cards Supplemental material, sj-docx-1-aut-10.1177_13623613241286025 for ‘Just knowing it’s there gives me comfort’: Exploring the benefits and challenges of autism alert cards by Chris Edwards, Abigail MA Love, Rebecca L Flower, Ru Ying Cai and Vicki Gibbs in Autism

sj-docx-2-aut-10.1177_13623613241286025 – Supplemental material for ‘Just knowing it’s there gives me comfort’: Exploring the benefits and challenges of autism alert cards Supplemental material, sj-docx-2-aut-10.1177_13623613241286025 for ‘Just knowing it’s there gives me comfort’: Exploring the benefits and challenges of autism alert cards by Chris Edwards, Abigail MA Love, Rebecca L Flower, Ru Ying Cai and Vicki Gibbs in Autism

## Notes

1. We have intentionally chosen to capitalise the term ‘Autistic’ to acknowledge its role as a significant and valued identity. This decision reflects the importance of recognising Autistic individuals as part of a cohesive and empowered community.
