# Supplementary figures and images for: ‘Just knowing it’s there gives me comfort’: Exploring the benefits and challenges of autism alert cards
Source: Autism. 2024 Oct 18;29(3):673–83. doi: 10.1177/13623613241286025 (PMC11894890; doi:10.1177/13623613241286025)

**Supplementary Materials**


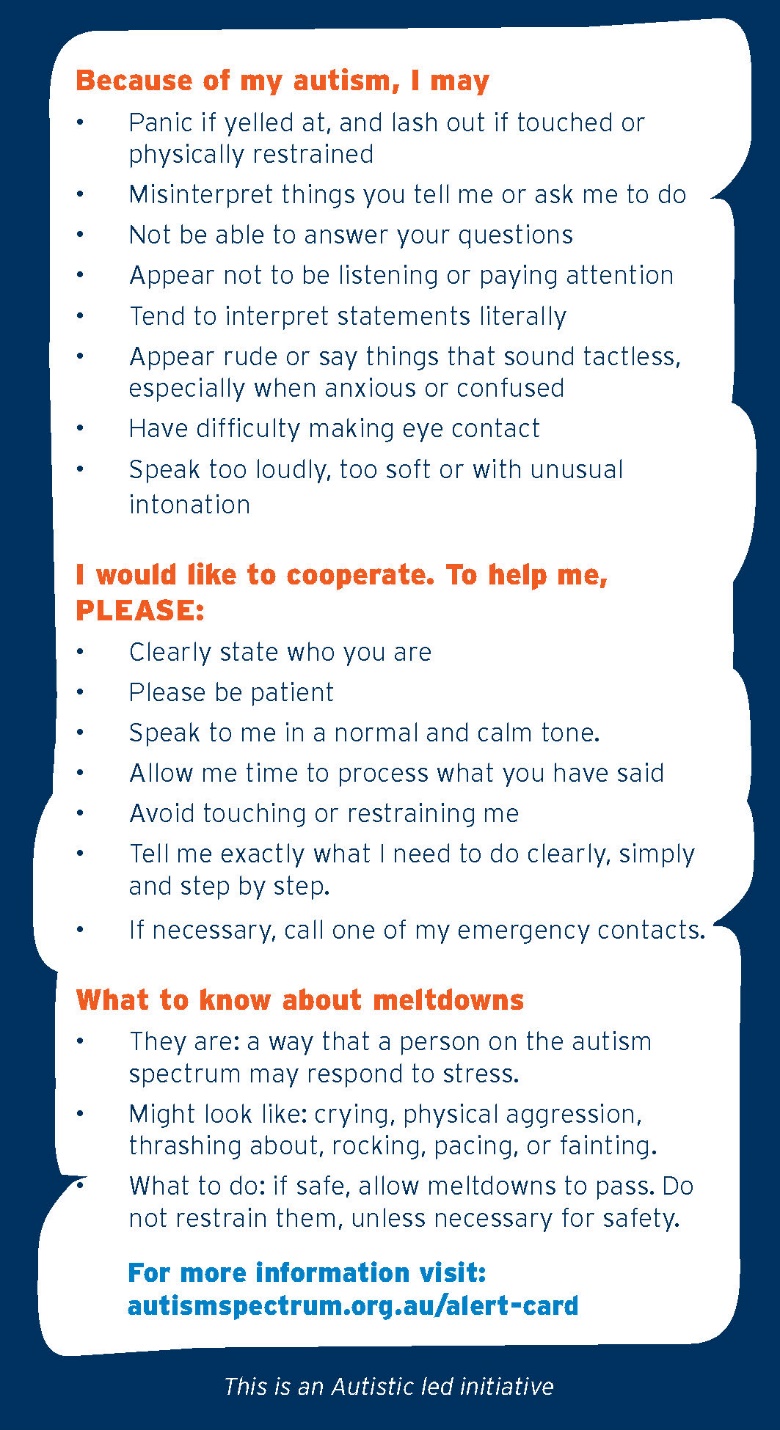

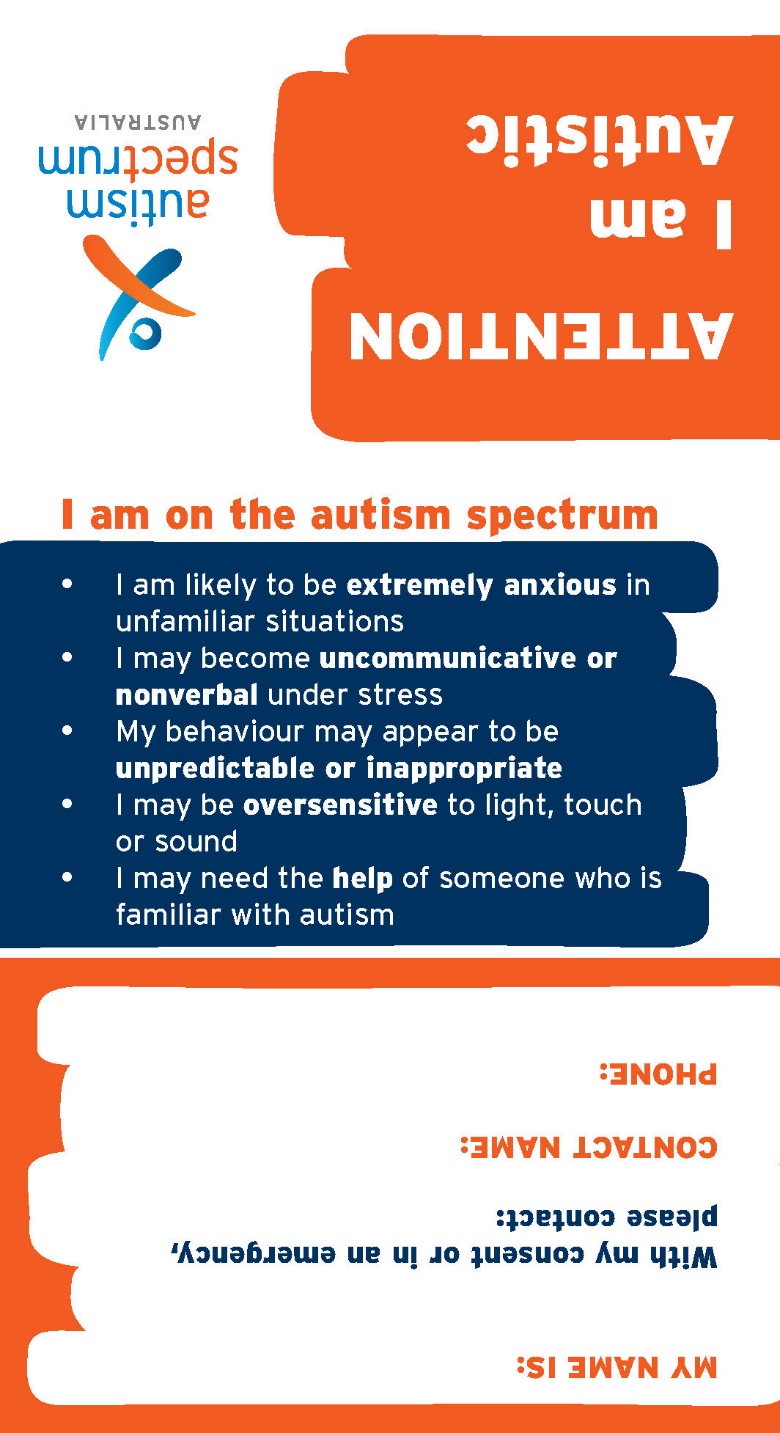
**Images of the Autism Alert Card**

Supplement: sj-docx-1-aut-10.1177_13623613241286025 – Supplemental material for ‘Just knowing it’s there gives me comfort’: Exploring the benefits and challenges of autism alert cards [file sj-docx-1-aut-10.1177_13623613241286025.docx]
