# Supplementary material for: ‘Just knowing it’s there gives me comfort’: Exploring the benefits and challenges of autism alert cards
Source: Autism. 2024 Oct 18;29(3):673–83. doi: 10.1177/13623613241286025 (PMC11894890; doi:10.1177/13623613241286025)
Supplement: sj-docx-2-aut-10.1177_13623613241286025 – Supplemental material for ‘Just knowing it’s there gives me comfort’: Exploring the benefits and challenges of autism alert cards [file sj-docx-2-aut-10.1177_13623613241286025.docx]

**Survey Questions**

1. About how long have you had the autism alert card? (provide your best guess): [text]
2. Where is the autism alert card kept? (you can select more than once): [in a wallet/purse/personal bag; in a home; in a vehicle; other (please specify); I don’t know]
3. Has the autism alert card been used?: [yes; no; I don’t know]
   - Yes: Who primarily uses the autism alert card?: [Autistic person; parent/caregiver; support person]
   - Yes: How often do you think the autism alert card is being used? (provide your best guess): [rarely (less than once a month); monthly; weekly; daily]
   - Yes: In what types of situations or settings has the autism alert card been used? (select as many as you like): [education; retail; community; home; workplace; healthcare; public transport; emergency services; other (please specify)]
   - Yes: Please provide a couple of sentences explaining why you used the autism alert card in this setting/these settings: [text]
   - Yes: What types of reactions have you encountered when using the autism alert card?: [mostly positive; mostly negative; mixture of positive and negative]
   - Yes: Can you elaborate on the reactions you have received when using the autism alert card?: [text]
   - No: Could you please specify why the autism alert card hasn’t been used?: [text]
   - No: Can you think of a situation where you would use the autism alert card? If so, please describe: [text]
4. What changes could be made to the autism alert card to make it more applicable or helpful for you?: [text]
5. How helpful has the autism alert card been for you?: [very helpful; helpful; neutral (neither helpful or unhelpful); unhelpful; very unhelpful]
   - Please explain why you think the autism alert card has been helpful/unhelpful?: [text]
6. Would you recommend Autistic people have an autism alert card?: [yes, I would recommend it; maybe/it depends; no, I would not recommend it]
   - Can you please share why you gave the recommendation you did?: [text]
7. Do you have any additional comments or feedback about the autism alert card?: [text]
